# Human state anxiety classification framework using EEG signals in response to exposure therapy

**DOI:** 10.1371/journal.pone.0265679

**Published:** 2022-03-18

**Authors:** Farah Muhammad, Saad Al-Ahmadi

**Affiliations:** 1 Department of Computer Science, King Saud University, Riyadh, Saudi Arabia; 2 Computer Science Department, College of Computer and Information Sciences, King Saud University, Riyadh, Saudi Arabia; Universiti Tunku Abdul Rahman, MALAYSIA

## Abstract

Human anxiety is a grave mental health concern that needs to be addressed in the appropriate manner in order to develop a healthy society. In this study, an objective human anxiety assessment framework is developed by using physiological signals of electroencephalography (EEG) and recorded in response to exposure therapy. The EEG signals of twenty-three subjects from an existing database called “A Database for Anxious States which is based on a Psychological Stimulation (DASPS)” are used for anxiety quantification into two and four levels. The EEG signals are pre-processed using appropriate noise filtering techniques to remove unwanted ocular and muscular artifacts. Channel selection is performed to select the significantly different electrodes using statistical analysis techniques for binary and four-level classification of human anxiety, respectively. Features are extracted from the data of selected EEG channels in the frequency domain. Frequency band selection is applied to select the appropriate combination of EEG frequency bands, which in this study are theta and beta bands. Feature selection is applied to the features of the selected EEG frequency bands. Finally, the selected subset of features from the appropriate frequency bands of the statistically significant EEG channels were classified using multiple machine learning algorithms. An accuracy of 94.90% and 92.74% is attained for two and four-level anxiety classification using a random forest classifier with 9 and 10 features, respectively. The proposed state anxiety classification framework outperforms the existing anxiety detection framework in terms of accuracy with a smaller number of features which reduces the computational complexity of the algorithm.

## 1 Introduction

Anxiety is a mental health problem that can cause serious damage to our bodies [1]. Anxiety is characterized by the feeling of restlessness, fear, and worry. Anxiety is the natural response of the body to a stressful scenario e.g., first day at school, before appearing for an interview or exam [2], prior to a public speaking task [3], due to a painful medical process [4] or a chronic life-threatening disease [5]. Every individual in society has to face anxiety in atleast one phase of life. Anxiety is a normal emotion and can be beneficial in many circumstances. Confronting an anxious situation triggers our natural fight or flight response resulting in the release of hormones, hence making us more vigilant and attentive to our surroundings. However, if the stressful setting continuous for a prolonged duration it can result into anxiety disorders which is harmful for the human health.

Human stress and anxiety have been used as interchangeable terms in the acedemic literature because they share many common symptoms like faster heartbeat and breathing, diarrhea, or constipation, but there exists a distinction between the two emotions. Stress is a sentiment of being unable to handle the psychological or emotional burden caused by an external stimulus. Some of the symptoms precisely linked with stress include anger, loneliness, dizziness, general unhappiness, and a feeling of being overwhelmed. The cause of stress can be short-term (e.g., a deadline at the office or a quarrel with a family member) or long-term (e.g., chronic illness, bad financial conditions, or an unhappy marriage). On the other hand, anxiety originates due to factors that are persistent and continues to exist even after the stressful situation is over. Symptoms explicitly connected with anxiety are restlessness, tension, sweating, nervousness, and a feeling of uneasiness. Chronic stress has been found to be a key factor in developing depression and anxiety symptoms with an effect on sleep, mood, and appetite of an individual [6–9].

Anxiety can be categorized into two main types i.e., state anxiety and trait anxiety [10]. State anxiety echoes the psychological and physiological response of a person to a particular situation at hand whereas, on the contrary, trait anxiety describes the personality trait of an individual. Human trait anxiety is commonly measured using rest state recording [11] whereas, state anxiety is an instant form of anxiety and is generally measured in response to some stimulus which instigates mental strain [12]. When a person is facing stress or anxiety, the human body responds by providing the required physiological and immune system adjustment in order to achieve the required cerebral equilibrium [13]. Stressors trigger changes in the human nervous system which instigates numerous physical and physiological variations in the human body which have been measured with different markers to date [14].

Human anxiety is a common mental health concern for a very large segment of the world population. According to the statistics of a survey conducted in the United States of America (USA), approximately 18% of the total population (i.e., 40 million people) is affected by an anxiety disorder and only 36.9% of these individuals from among these anxiety patients are receiving treatment [15]. Moreover, according to the findings of the Anxiety and Depression Association of America (ADAA), the probability of getting hospitalized is six times higher in anxiety patients as compared to a healthy individual [16]. According to another survey conducted by the National Institute of Mental Health, 19% of the American individuals over 18 years of age experience an anxiety disorder and approximately 31% of the USA residents experience stress throughout their lifetime [17]. Prolonged anxiety disorder weakens the immune system of a person and hence the likelihood of cardiovascular diseases is strikingly increased. A strong bond between anxiety disorder, stroke, and heart disease has been reported in a number of research studies [18, 19]. Anxiety disorder tends to develop a diverse type of risk factors which include depression, genetic problems and personality disorders. Approximately half of the anxiety disorder patients tend to face depression depicting a strong association between the two mental health issues. Anxiety disorder can be categorized into generalized anxiety disorder (affecting 3.1% of the U.S. population), panic disorder (affecting 2.7% of the U.S. population), social anxiety disorder (affecting 6.8% of the U.S. population), Obsessive-Compulsive Disorder (affecting 1.0% of the U.S. population), and Post-traumatic Stress Disorder (affecting 3.5% of the U.S. population). According to statistics, women are more prone to generalized anxiety disorder, panic disorder, obsessive-compulsive disorder and post-traumatic stress disorder whereas social anxiety disorder is faced equally by both genders. In addition to the adults, 25.1% of the children, between the age of 13 to 18 years, are also victims of anxiety disorder. Researchers have reported that children with anxiety disorder have poor performance at school, miss out on important social experiences and become drug addicts [20, 21]. Evasion of stressful situations is of no benefit to human health nor is it helpful in devising a strategy to cope with these anxious state of affairs in the future. The above mentioned statistics illustrates the fact that assessment of human anxiety is of supreme importance and appropriate measure to lessen the consequences of anxiety to human health need to be developed.

Diagnoses of anxiety have been a challenge both for researchers and clinicians. Clinical analyses mainly depend on the documented symptoms frequently detected in the anxiety patients but there exists a significant overlap in these diagnostic criteria for stress, anxiety, and depression [22]. It is possible that two individuals spotted with anxiety and depression may share a common symptom. This symptoms-based diagnostic method can help medical practitioners in giving their verdict, but this approach lacks in providing a quantifiable objective measure from which fundamental reasons for the occurrence of anxiety can be sensed. It can be deduced from these facts that there exists a clear gap in the clinical domain to develop an impartial and accurate measure to detect anxiety and its disorders. The biomedical research community believes that chemical biomarkers like salivary alpha-amylase and chromogranin A are the more trustworthy solutions for the anxiety assessment [23, 24].

Moreover, the psychologists consider the standard psychometric questionnaires as a dependable measure of anxiety. These questionnaires have been used for quite a long time and have provided an accurate assessment of human anxiety, but they are not able to provide any insight into the physiological changes occurring within the body of the individual affected by the anxiety. Some of the regularly used questionnaires for the quantification of anxiety include State-Trait Anxiety Inventory (STAI) questionnaire [25], Hamilton Anxiety Measure (HAM-A) [26], Depression, Anxiety and Stress Scale (DASS) [27], Death Anxiety Questionnaire (DAQ) [28], Beck Anxiety Inventory (BAI) [29] and Scale of Death Anxiety (SDA) [30]. Another incipient and imperative method of valuation of anxiety is the use of physiological constructs. Anxiety measurement has been achieved by using electroencephalography (EEG) [31], electrocardiography (ECG) [32], photoplethysmography (PPG) [33], galvanic skin response (GSR) [34], heart rate (HR) [35], heart rate variability (HRV) [8], blood pressure (BP) [36], and electromyography (EMG) [37] signals. In this study we aim to develop an EEG signals-based state anxiety measurement framework because of its importance in curing individuals with anxiety disorders and helping them become useful segment of the society. We intend to identify the relationship between the EEG recording headband electrodes, EEG frequency bands and the anxious and non-anxious individuals.

## 2 Literature review

A diverse range of human anxiety measurement schemes using physiological signals has been proposed in the literature which can be characterized into two lines i.e., state-anxiety and trait-anxiety. The footsteps for the general framework of human anxiety quantification are outlined beneath. Firstly, the physiological signals of the participants are acquired in the rest-state (for measurement of trait anxiety) [38] or in response to stimulus (for the measurement of state anxiety) [12]. Subsequent to data acquisition, is a feature set that has the ability to segregate anxious and non-anxious subjects is extracted. Finally, an appropriate classification algorithm is employed to quantify human anxiety from the recorded physiological signals.

Human state anxiety detection based on features extracted from the EEG signals recorded in response to face-to-face psychological stimuli is presented in [39]. The study reported an accuracy of 86.7% for the four-class anxiety classification using a stacked sparse autoencoder scheme. An association between the math anxiety and the EEG signals of the participants is discussed in [40] with a reported accuracy of 93.75% using the Naïve Bayesian tree. A new EEG based anxiety detection and mitigation massage headband is developed in [41]. Beta waves have been found to be associated with the high anxiety participants whose amplitude was successfully suppressed by the massage headband. Another state anxiety measurement scheme using EEG signals in response to emotional video clips is presented in [31]. The study discovered the fact that asymmetry index values were higher in relaxed subjects and are reduced during the anxious situation. An EEG-neurofeedback scheme for anxiety classification in response to mindfulness recording and resting-state EEG signals is presented in [42]. A visual analogue scale was used to subjectively label the participants into three anxiety classes. The highest accuracy of 87.18% for anxiety classification was achieved using the SVM algorithm.

Another type of state anxiety i.e., social anxiety is measured using heartbeat signals recorded in response to two different social evaluative situations i.e., public speaking and thesis defending in a study conducted in [43]. Statistical analysis of baseline and anxiety state data revealed that the complexity of the heartbeat is significantly reduced during social anxiety situations. Moreover, accuracy of 81.82% is attained for detection of low and high anxiety state using support vector machine (SVM) classifier. Another state anxiety measurement scheme using heartbeat intervals of student recording prior to the university examination is elaborated in [35]. Statistical analysis of the data revealed that a negative correlation was found between state anxiety and the largest Lyapunov exponent as well as entropy measure feature. The relationship of PPG data with the state anxiety is explored in [33]. A strong correlation of 0.81 between the subjective score of the participants and the anxiety detected by the machine learning-based approach is attained.

An objective anxiety measurement framework using the fusion of features extracted from the EEG and the PPG signals recorded in response to two scenarios i.e., riding a bike at their most comfortable speed and while imagining that they had to contend with somebody who is riding at 80km/hr. is discussed in [44]. Principal component analysis and k-nearest neighbor classifier led to an accuracy of 62.5% for three state anxiety classification. A wearable sensor-based state anxiety detection study in two and three levels using physiological signals of ECG, GSR, and respiration rate acquired in a randomized controlled trial is presented in [45]. An accuracy of 89.8% and 74.4% is attained for two and three-class anxiety classification using a bagged tree classifier, respectively. A state anxiety identification scheme in older individuals with ages ranging from 60-80 years using physiological signals of GSR and PPG and a context-based feature in response to trier social stress test (TSST) protocol is explored in [46]. Ground truth for the proposed scheme was obtained using STAI questionnaire responses from the participants. Results of the study showed that with the addition of context-based features, a 3.37% and 6.41% higher accuracy is obtained by the random forest classifier in comparison to only using GSR and PPG signals, respectively. An anxiety detection framework using eye, mouth, and head movement activity and heart rate feature through video recorded facial cues is discussed in [47]. The highest classification accuracy of 91.68% is achieved for anxiety detection using the AdaBoost classifier. Driver’s anxiety detection using a fusion of EEG, PPG, GSR, and pupil size of the subject is examined in [48]. EEG signal resulted superior in performance with an accuracy of 77.01% in comparison to the fusion of other signals which resulted in lower accuracy as compared to EEG. A four-level anxiety level recognition scheme for virtual reality therapy system using physiological signals of BP, GSR, and skin temperature is elaborated in [49]. An accuracy of 80.1% is achieved by the SVM classifier using the leave one-subject out cross-validation scheme. A stress and anxiety detection scheme in the academic environment using physiological signals of heart rate, skin temperature and oximetry signals is presented in [50]. The highest classification accuracy of 95% was achieved for two level anxiety classification using SVM classifier with a feature vector length of 3. Anxiety-displaying activities recognition study using motion sensors of accelerometer, gyroscope and magnetometer is presented in [51]. A deep learning model consisting of Convolutional Neural Network and Long Short Term Memory achieved an accuracy of 92% for anxiety related activities.

Human state anxiety assessment schemes available in the literature have used a diverse range of anxiety-inducing stimuli’s (Trier Social Stress Test (TSST) [52], Cold Pressor Test (CPT) [53], public speaking [54, 55] and mental arithmetic task [56]) and physiological signals (EEG, ECG, GSR, PPG, HR, and BP) to quantify anxiety in a robust manner. However, the body of the literature on human anxiety measurement is still growing and is not as rich as the number of studies available for human stress measurement. There exists a significant scope to further strengthen the findings of the existing anxiety assessment schemes. Recently, a new publicly available dataset for the assessment of human state anxiety using EEG signals named “A Database for Anxious States based on a Psychological Stimulation (DASPS)” has been developed by the authors in [12]. The authors have investigated the trial duration, feature type, feature combination, and anxiety levels of the participants in their study conducted in [39]. The study determined the fact that 1 second time duration is optimum for anxiety stimulation and achieved an accuracy of 86.7% for four-level anxiety classification. However, the proposed scheme has not examined the effect of EEG electrodes and frequency band selection for the optimum classification of anxiety into multiple levels. Moreover, the selection of the best subset of features from the selected EEG frequency band is still pending and needs to be inspected to improve the computational complexity of the algorithm. To this end, we propose a novel anxiety classification model addressing the above-mentioned challenges of the channel, EEG frequency band, and feature selection in the DASPS dataset to improve the accuracy and the robustness of the anxiety detection algorithm. The recorded EEG signals in the DASPS are processed for noise cancellation and channel selection in the pre-processing phase. Following this, the frequency bands of the EEG signal are computed using discrete-time Fourier transform, and feature extraction is performed in the frequency domain. EEG frequency band selection algorithm is applied to select the optimum frequency band for the anxiety classification. Furthermore, the extracted features from the selected EEG frequency bands are applied upon with a feature selection algorithm to further improve the computational complexity of the proposed scheme. Finally, the selected subset of features from the optimum EEG frequency band of the statistically significant EEG electrodes are subjected to classification using five different classification algorithms which include k-nearest neighbors (kNN), decision tree (DT), random forest (RF), multilayer perceptron (MLP), and SVM. Moreover, another benefit of using the DASPS dataset for anxiety quantification is that the EEG signals are acquired using a low-cost commercially available headset which can be used for anxiety detection in both laboratory and out of laboratory environment.

### 2.1 Our contribution

The main contribution of the proposed anxiety classification scheme as compared to the existing research is elaborated below.

1. Firstly, all the existing state anxiety classification schemes that have been developed using the EEG datasets which are not publicly available for future research and hence cannot be used to further improve the results of their proposed scheme. A fair comparison is not possible with their methods, whereas, in our proposed scheme a publicly available EEG signals database named DASPS” has been used and we have worked on improving the results of the study in which the DASPS database has been developed.
2. Secondly, we have examined the effect of EEG electrodes and frequency band selection for the optimum classification of anxiety into multiple levels.

To the best of our knowledge, only one existing study discussed in [57] has explored the effect of these parameters (electrode (channel) and frequency band selection of the EEG data) for state anxiety classification. There are some shortcomings of this existing anxiety classification scheme as compared to our proposed scheme which include low classification accuracy for four level anxiety classification and a bulky feature vector length. The proposed scheme addresses these concerns by exploring the effect of selecting optimum subset of features from the selected EEG frequency band to lessen the computational complexity of the algorithm. The earlier schemes have employed feature selection from the entire EEG data without selecting optimum subset of EEG electrodes and frequency bands.

The organization of the rest of the paper is as follows. The proposed human anxiety quantification framework comprising of details about data acquisition protocol, pre-processing, feature extraction, frequency band, and feature selection, and classifiers adopted for the anxiety classification is elaborated in Section 3. Section 4 focuses on the experimental results and their analysis followed by the comparison of the proposed algorithm with the state-of-the-art schemes available in the literature. Finally, the conclusion of the proposed scheme is outlined in Section 5 of the paper.

## 3 The proposed anxiety detection framework

**Fig 1:**
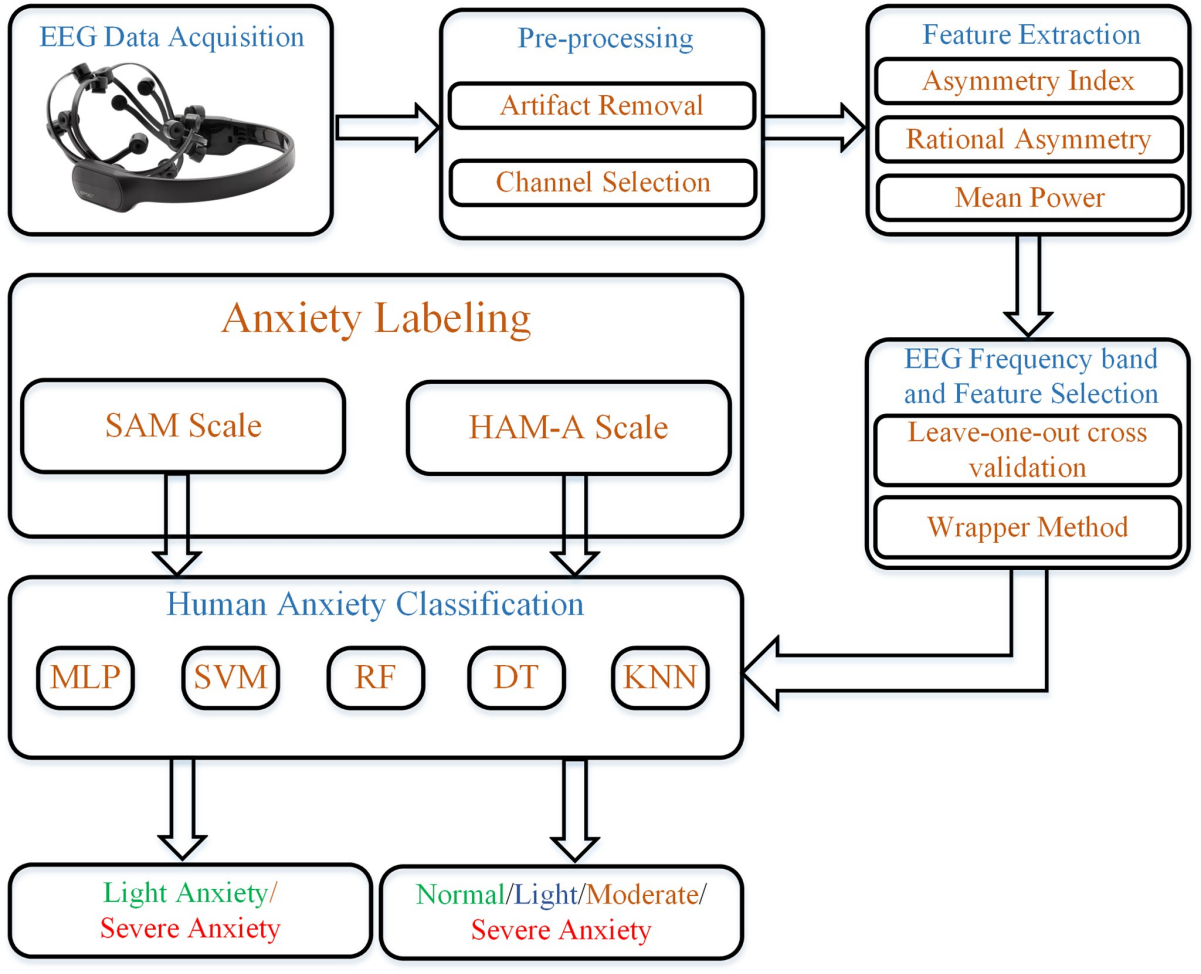
Block diagram of the proposed human anxiety classification scheme in response to exposure therapy.

The proposed anxiety detection framework is presented in Fig 1. The main slabs of the scheme consist of experimental protocol description, pre-processing, feature extraction, EEG frequency band, feature selection, and classification. Details about each of the blocks are expounded in the following sub-sections.

### 3.1 Experimental protocol

In this study, the DASPS database consisting of EEG signals recorded in response to exposure therapy is used. Exposure therapy is a popular type of Cognitive Behavioral Therapy (CBT) that involves stating situations that prompt anxiety to a level that is both comfortable and tolerable [58]. The details about the EEG database are explained in this section. EEG signals of 23 healthy participants (10 males, 13 females) with no reported history of any mental or physical illness having an average age of 30 years were recorded during the experiment. The involvement of the participants in the experiment was entirely voluntary. A written consent was obtained from each subject prior to the start of the experiment. (As we have used a publicly available EEG dataset in our study, which the authors have developed in [39] but they have not mentioned the name of ethics committee which approved the experimental protocol rather they have mentioned that the written consent was obtained from all the participants.) Next, the participant is asked to subjectively report their anxiety level before the experiment using Hamilton Anxiety Rating Scale (HAM-A). The tool is composed of 14 different items, where each item corresponds to a number of symptoms and can be rated on a scale of 0 to 4.

**Fig 2:**
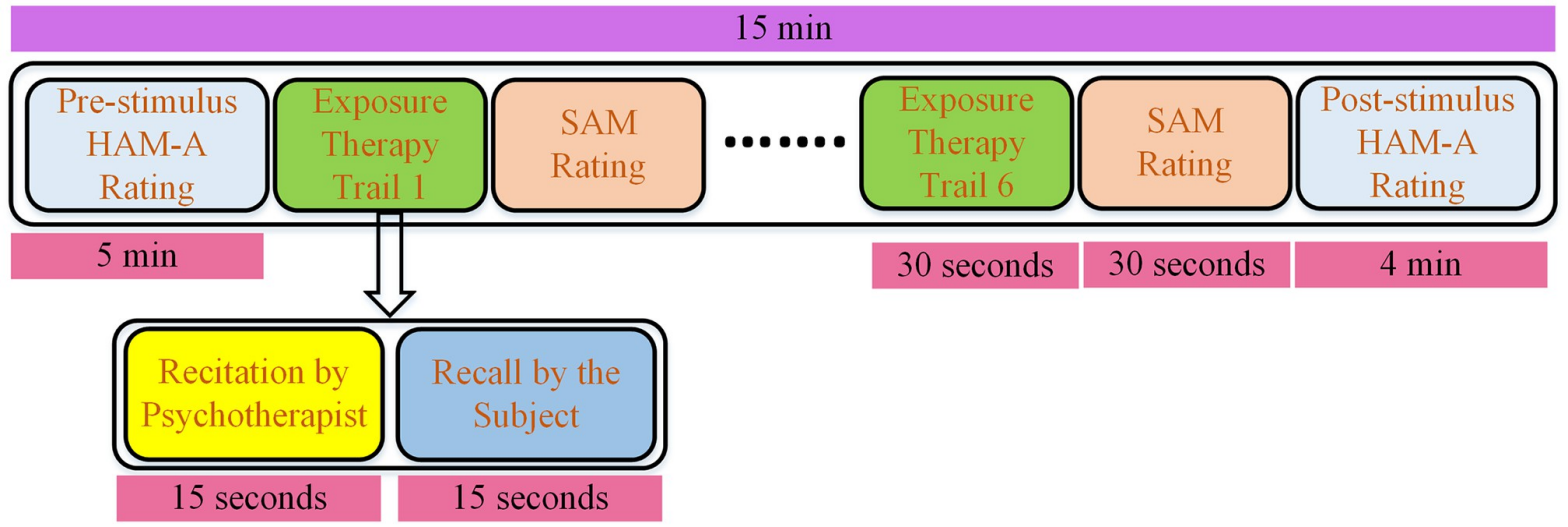
Timing diagram of the data acquisition protocol followed in DASPS database.

The total score of the questionnaire can range from 0 to 56. This prior anxiety severity computation helps the psychotherapist to determine the amount of exposure a particular person can withstand. Following the subjective anxiety assessment, the subject is asked to be seated in a comfortable chair in a closed eye manner avoiding speech and gesture movements as much as possible. The psychotherapist starts the recital of the first scenario and aids the subject of the experiment to imagine it to elicit anxiety. Both the recital and the imagination phase are composed of 15 seconds each. The Self-Assessment Manikin (SAM) scale is used to inquire the subject that how he felt during the stimulation. SAM scale consists of two parameters i.e., valance which ranges from negative to positive, and arousal that runs from calm to excited state. Both valance and arousal have a nine points scale for measuring the subject’s emotion. This sequence of provoking anxiety by psychotherapists and evaluation of valance and arousal by the subject is executed six times in the experiment. Finally, at the end of the experiment, HAM-A was once more answered by the subject to report his current anxiety level. After this, the participant was allowed to leave the experiment room. The timeline diagram for the experimental protocol is presented in Fig 2.

#### 3.1.1 EEG recording equipment

EEG data acquisition is performed using Emotiv EPOC wireless headset. The headset consists of 14 electrodes and 2 mastoids placed according to the 10-20 electrode positioning system [59]. The electrodes included in the Emotiv EPOC EEG headset are AF3, F7, F3, FC5, T7, P7, O1, O2, P8, T8, FC6, F4, F8, and AF4. One of the mastoids acts as a ground reference point to compare the voltage of all other electrodes of the headset whereas, the other mastoid is used as an indirect reference to minimize the effect of electrical interference [60]. Some of the salient features to use the Emotiv EPOC headset in this experiment include ease of use, the faster setup time of 3 to 5 minutes, rechargeable battery which lasts up to 12 hours. Moreover, due to the wireless nature of the headset, it is easy to wear for the user and does not require a complex setup like the clinical EEG data acquisition equipment. The efficacy of the Emotiv EPOC headset has been reputable in a number of emotion recognition [61–66] and stress measurement studies [67–70] available in the literature.

The reliability of the Emotiv EPOC headset as compared to medical grade EEG data acquisition equipment has been explored in several studies in the literature to validate the findings of the consumer grade EEG devices. In the studies conducted in [71] and [72], the authors have conducted an experiment related to auditory event-related potentials (ERPs) to find out the equivalence of the quality for the ERPs measured using consumer grade Emotiv EPOC headband and medical grade Neuroscan EEG equipment in adults and children, respectively. The studies concluded the fact that the consumer grade EEG system can be reliably considered a valid alternative to medical grade devices for the acquisition of EEG signals. In addition to this, another experiment conducted in [73] in which the authors tried to identify the quality of the signals of two different consumer grade EEG data acquisition equipment’s for control tasks. The authors deduced the fact that the Emotiv EPOC headset has been substantially better as compared to the other consumer grade device for the classification of variety of control tasks. Moreover, the Emotiv EPOC headset has been used in a range of emotion recognition studies like evaluation of music pleasure [74] and stress [68] with good level of reliability and accuracy. Our study is focused on measurement of state anxiety which is also an emotion that can be reliabily estimated via Emotiv EPOC headband.

**Fig 3:**
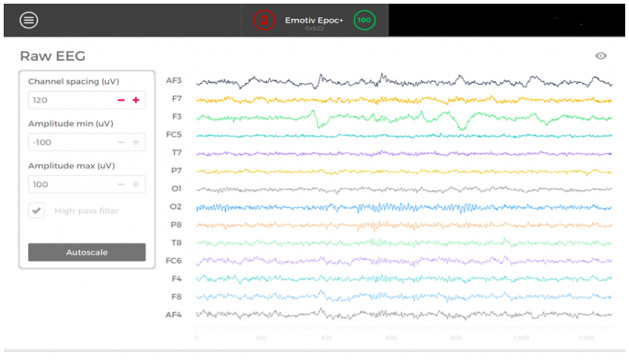
Raw EEG data obtained from the 14 channels of the Emotiv EPOC headset.

Emotive EPOC headset records the raw EEG data for all the 14 electrodes and saves it in the form of an “.edf” file which can be converted to a “.csv” or “.mat” file for further processing. EEG data were recorded for a duration of 6 minutes with each trial consisting of 1 minute which is composed of 30 seconds for the recitation of the situation by psychotherapist and imagination by the subject and 30 seconds for answering the SAM rating scale. The sampling rate of the EPOC headset is 128 Hz and the impedance was kept at 7 kilo-ohms. Raw experimental output for the EEG signal acquired from the Emotiv EPOC headset is shown in Fig 3.

#### 3.1.2 Data analysis

In order to analyze the effect of stimuli in eliciting anxiety, the authors in [12], analyzed the SAM score after every trial of the experiment and HAM-A scores of the subjects at the start and end of the experiment. Russell explained the concept of anxiety as a situation corresponding to low valance and high arousal (LVHA) in a study conducted in [75, 76]. Hence for the measurement of anxiety, the quadrant of LAHV is the main region of focus. To evaluate the relative variability of the participant’s scores for all the six trials of the situation, the Coefficient of Variation (CV) was computed. A mean CV of 0.58 and 0.42 was obtained for valance and arousal score across all the participants for each trial of the experiment which can be regarded as a low variance. The authors have also reported that when the valance and arousal score of the participants was plotted on a 2D plane consisting of Low Valence and Low Arousal (LVLA), High Valence and Low Arousal (HVLA), Low Valence and High Arousal (LVHA), and High Valence and High Arousal (HVHA) quadrants, the majority of the participants were found in the LVHA and LHLA quadrant advocating the fact that the stimuli used was effective in provoking anxiety in most subjects. Moreover, it was also reported that the number of subjects having severe anxiety before the experiment was 7 whereas after facing the stimulus the number of participants augmented to 13 again stressing the fact that stimuli was efficacious in anxiety elicitation.

### 3.2 Pre-processing

In biomedical signal processing, it is necessary to pre-process the data to suppress the noise and artifacts from the acquired signals so that a clean signal is reached which could be fed to the feature extraction stage to realize decent classification accuracy. EEG signals contaminated by the physiological artifacts generated by parts of the human body other than the human brain include electrooculography (EOG) artifacts (having frequency range less than 4 Hz) and electromyography (EMG) artifacts (having frequency range greater than 30 Hz). Moreover, other than the physiological artifacts generated by different parts of the human body, there also exist artifacts caused by the power line interference having a frequency range of around 50 Hz.

#### 3.2.1 EEG artifact removal

EOG artifacts can be subdivided into eye blinking and eye movement artifacts. Both types of artifacts belong to low frequencies i.e., less than 4 Hz, but eye blinking artifact has significantly higher amplitude as compared to eye movement artifact. Moreover, the eye blinking artifacts are mainly identified in the EEG data from the frontal electrodes of the headband whereas, on the other hand the eye movement artifacts are characterized by the difference in the symmetry of the left and right brain hemisphere. EMG artifacts can be categorized in two ways i.e., forehead movement and jaw clenching artifacts. Both forehead movement and jaw clenching are primarily an activity consisting of high frequency components i.e., less than 13 Hz with some exception of low frequencies. The presence of these frequencies can significantly deteriorate the diagnosis criterion and can lessen the usefulness of the recorded EEG data significantly. Therefore, these artifacts need to be eliminated from the EEG data to make the diagnoses more effective and reliable. In addition to applying the artifact removal techniques the participants in the experiment were instructed to keep muscles and eye movement minimum to achieve good quality EEG data.

To remove EOG and EMG artifacts from the recorded EEG signals, an automatic artifact removal tool of the EEGLab [77] was employed. An appropriate finite impulse response (FIR) bandpass filter having passband ranging from 4 to 45 Hz (order = 15, filter length = 16, window = Hanning) to truncate the baseline, muscular and ocular artifacts from the acquired signals was applied. Moreover, a cutoff frequency of 45 Hz ensures that power line noise interference is also mitigated from the EEG signal. To conquer the effect of EMG noise from the signal, blind source separation canonical correlation analysis-based algorithm implemented in EEGLab was used [78]. The algorithm used a criteria *emg_psd* which defines a threshold for the average power ratio of the recorded EEG signal and the components which have a value less than the defined threshold are regarded as EMG artifacts and are discarded. The estimator used for the valuation of power spectral density of the EEG signal is hamming window-based Welch periodogram. For the removal of EOG artifacts, the EEGLab uses improved weighted adjusted second-order blind identification method (iWASOBI) and fractal dimension parameter [79]. As a rule of thumb, the signal components having low fractal dimension values corresponds to low-frequency segments of the signal and is a suitable criterion for the identification of EOG components.

In the second phase of the pre-processing stage, channel selection is performed by application of student t-test and analysis of variance (ANOVA) for two and four-level, respectively on the EEG data obtained from Emotiv headset. Power spectral density is computed using the Welch algorithm with an overlapping window of 50%. The decision about statistically significant channels of the Emotiv EPOC headset is made on the basis of p-value and the channels are considered statistically significant if the p ≤ 0.05. Filtering and signal processing implementation for the proposed anxiety classification scheme are available at https://bit.ly/31ARYYo.

### 3.3 Feature extraction

In this study, the analysis of the recorded EEG data is performed in the frequency domain by computing the power spectral density of the raw EEG signals. Power spectral density is realized using the Welch method of the *Brainstorm*3 toolbox in MATLAB. Welch method uses an overlapping window of 50% for computing power spectrum followed by the application of hamming window to each of the overlapped sequences. Next to this, the Fast Fourier Transform (FFT) of each segment was computed and the power of the FFT coefficients of all overlapping windows was averaged out. Hence the raw EEG data is represented by the power spectrum of its frequency bands [80]. Obtained frequency bands of the EEG signal from each channel of the Emotiv EPOC include delta (2-4 Hz), theta (5-7 Hz), alpha (8-12 Hz), beta (13-29 Hz), and gamma (30-45 Hz) bands. Following this, the feature extraction is performed from these frequency ranges.

The reason for using fast fourier transform (FFT) as a feature extraction method over discrete wavelet transform (DWT) are manifold. Firstly, the computational power required to compute the DWT is higher as compared to the FFT algorithm, thus creating computational overhead. Secondly the additional information provided by the DWT over FFT is the time at which a particular frequency occurred in the EEG signal and in this study, we are not interested in locating the events in time, therefore use of DWT instead of FFT only adds to the computational cost. Moreover, when choosing FFT in comparison to discrete cosine transform (DCT) for feature extraction is due to the reason that FFT considers the harmonically related complex exponential functions whereas, DCT only takes real valued cosine functions. Therefore, DCT is more suited over FFT for applications where compression is needed.

Frequency domain features extracted from the EEG data of the selected channels of the Emotiv EPOC headset include rational asymmetry (RASM), mean power (MP), and asymmetry index (AI). RASM is the ratio of the power spectrum for the selected asymmetric channels of the left and right cerebral hemispheres. MP feature is represented by the average of the power spectrum of each band for each selected channel of the Emotiv EPOC headband.

### 3.4 EEG frequency band and feature selection

After extraction of the features from the selected EEG channels, the next phase is to apply a frequency band selection algorithm to select the optimum frequency band for the classification of human anxiety. To this end, we have applied the frequency band selection algorithm proposed in [81] to our EEG data. The frequency selection algorithm works by selecting the EEG frequency band which achieves the highest classification accuracy from among all the frequency band combinations. The algorithm iterates this procedure 1000 times to select the optimum frequency band. A combination of features from the theta and beta band of the EEG data resulted in the highest classification accuracy among all frequency band combinations.

After EEG frequency band selection, the features from the selected EEG frequency bands are applied upon with the wrapper method for feature selection to select the optimum subset of features. The wrapper method is a recursive feature elimination technique that removes the features which are least correlated with the class labels.

Recursive feature reduction is a highly efficient optimization technique that extracts the feature subset yielding the highest accuracy. Like any recursive function, this technique calls itself again and again keeping track of the best and the worst performing feature subsets and removing the worst-performing feature subset at the end of the iteration. In the next iteration, the feature selection is performed only with the remaining feature subset. This process continues recursively till the point reaches when there is no change in the feature subset in the subsequent iterations and the algorithm converges resulting in the final optimum subset of features.

### 3.5 Human anxiety classification

The feature vector obtained after EEG frequency band and feature selection is subjected to classification step via different machine learning techniques. A variety of machine learning classifiers with different sets of optimization parameters were used for our proposed anxiety classification scheme. The parameters used for the classification models were chosen using grid search hyperparameter tuning method. KNN is termed as a lazy learning algorithm because it stores all the examples of the training set and computes the distance of the test sample from each example of the training data and the test data is assigned to the class having a smaller Euclidean distance. For the KNN classifier we performed the grid search using values of k = 1,3,5,7,9 but the highest classification accuracy was obtained using k = 5. DT is another non-parametric supervised machine learning technique that is based on entropy measure of the data and it classifies the data by building a tree and predicting the output variable for the test data by simple decision rules deduced from the data features. Similarly, for the decision tree classifier we tried different depth values for the tree and found the depth value of 32 to be the optimum. Increasing the tree depth further does not improved the classification performance therefore a tree depth of 32 is used in the current study. RF is an extension of the DT classifier in which we have multiple decision trees (hundred or even thousands of them) and the final classification result is achieved by majority rule i.e., the final decision is the decision of the majority of the decision trees. For the random forest classifier, we used different number of decision trees and a value of 500 trees with a depth of 32 was found to be the best among all and hence it is used in this study. MLP is a back-propagation feed-forward neural network. The simplest kind of MLP network consists of single input, hidden, and output layers. An activation function that transforms the input to the output is implemented in the hidden layer of the network. The Multilayer perceptron classifier was tested with different optimizer and activation functions and the optimum classification results were obtained using 4 hidden layers, ‘Adam optimizer’ and ‘Relu activation function’ with a momentum and learning rate of 0.3 and 0.4, respectively. SVM is one of the most well-known algorithms used for supervised machine learning. It classifies the data points by building an N-dimensional hyperplane between the two classes. Radial basis function (RBF) produced the best results and hence is used as a SVM kernel in this study. In the proposed anxiety classification scheme, we have reported the classification results obtained from the best tuned parameters. The recently proposed deep learning methods were not used for the current study because the number of subjects involved in the study were only 23 and therefore the available EEG data was not sufficient to train the deep learning model. In future we intend to acquire EEG data from a larger number of subjects to be able to obtain results for anxiety classification using deep learning models.

## 4 Experimental results

This section presents the anxiety classification result of both two and four levels based on the selected subset of features from the optimum frequency band combination for the statistically significant channels of the Emotiv EEG headset. The analysis is performed using a trial duration of 1 second which was found to be the most optimum for anxiety classification in the studies conducted in [12, 39].

### 4.1 Labeling

The labeling of the subjects is performed in two different manners i.e., based on SAM rating and HAM-A scale. For the SAM rating-based labeling, trials of the experiment have valance < 5, and arousal > 5 are labeled as Normal whereas, the trials with valance scores in the range of 0 to 2 and arousal score in the range of 7 to 9 are labeled as Severe. Moreover, the subjects having valance scores between 2 to 4 and arousal scores between 6 and 7 are labeled as having Moderate anxiety, and the subjects with valance scores in the interval of 4 to 5 and arousal scores in the interval of 5 to 6 are labeled as having Light anxiety. The number of instances based on SAM-based labeling for a trial duration of 1 second for two-level anxiety detection are Light Anxiety: 2640 and Severe Anxiety: 1500. Similarly, the number of instances for four-level anxiety detection are Normal Anxiety: 2340, Light Anxiety: 300, Moderate Anxiety: 150, and Severe Anxiety: 1350.

**Fig 4:**
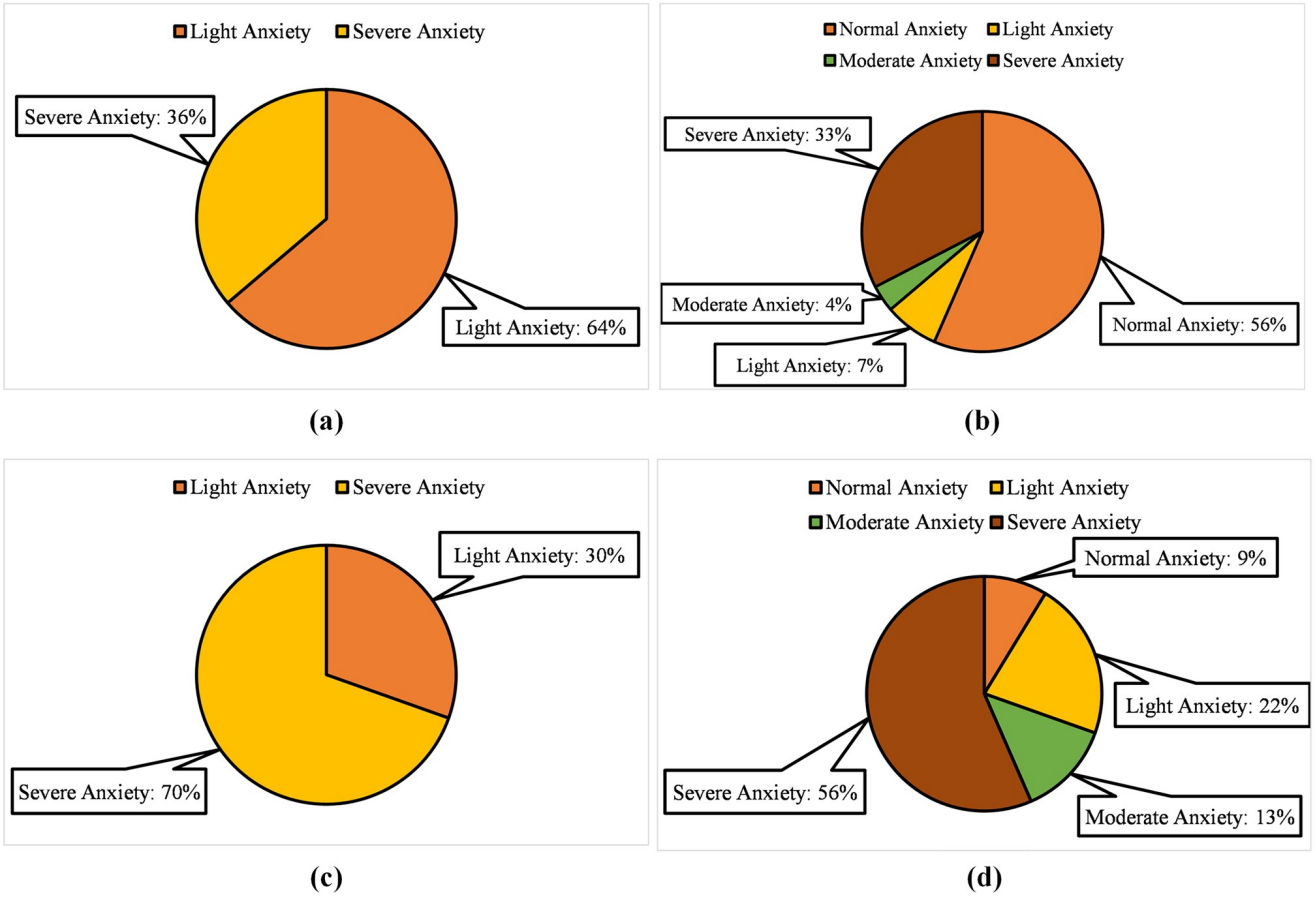
Percentage of instances using SAM score based labeling for (a) Two-level (b) Four-level and HAM-A based lableling for (c) Two-level (d) Four-level anxiety classification.

For labeling based on HAM-A based scale recorded at the end of the experiment, trials having HAM-A scores between 0 to 12 are labeled as normal anxiety, trials with scores between 12 to 20 are termed as having light anxiety. Moderate anxiety ranges between 20 to 25 and severe anxiety is labeled above the score of 25. Based on this labeling the number of instances for a trial duration of 1 second for two-level anxiety detection are Light Anxiety: 1260 and Severe Anxiety: 2880. Similarly, for four-level anxiety detection, the number of instances are Normal Anxiety: 360, Light Anxiety: 900, Moderate Anxiety: 540, and Severe Anxiety: 2340. Fig 4 shows the number of instances of each anxiety level using both SAM and HAM-A based labeling.

### 4.2 Channel selection

**Fig 5:**
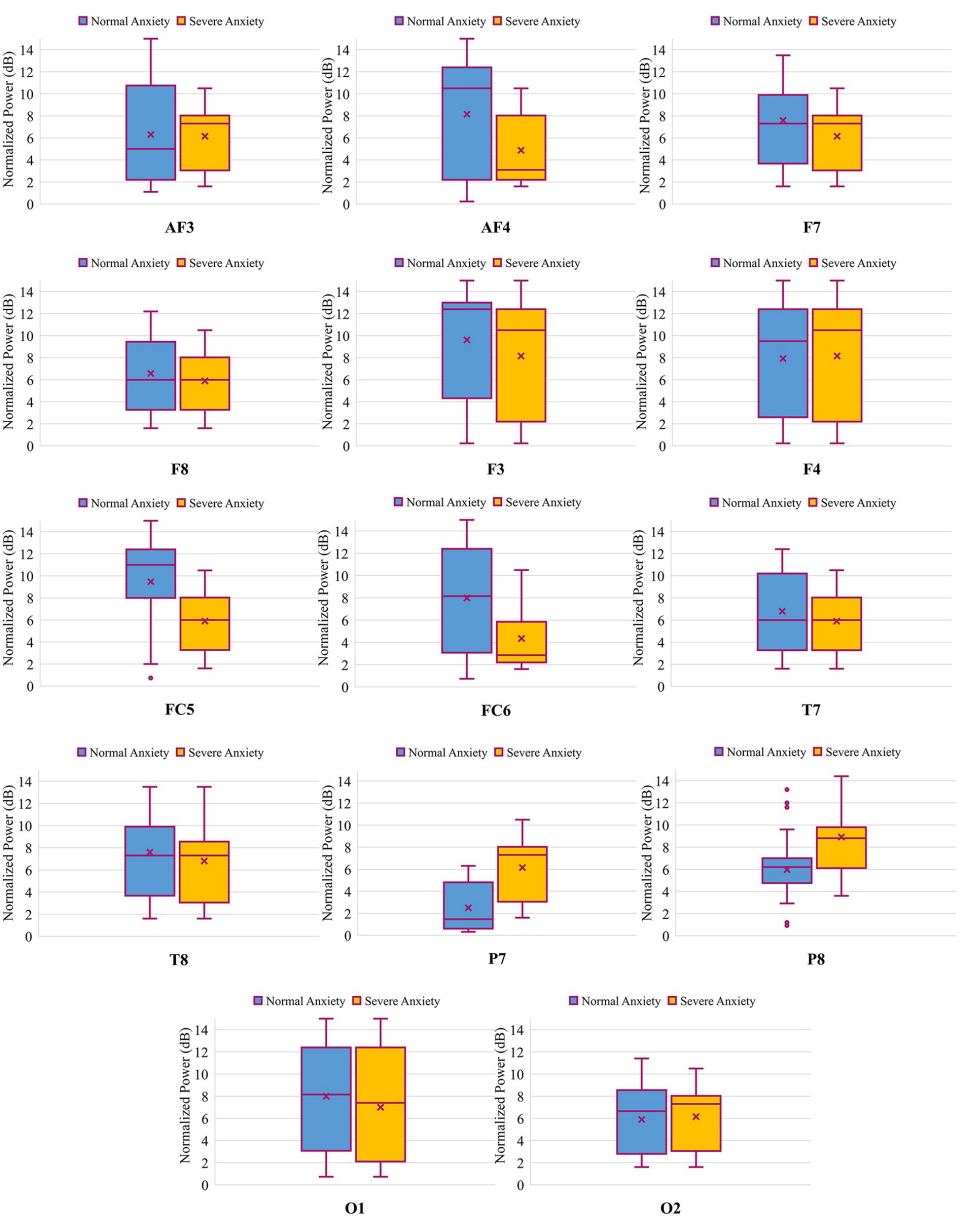
Box-and-whisker diagram of the power obtained from each channel of the Emotiv EPOC headset for binary anxiety classification.

**Fig 6:**
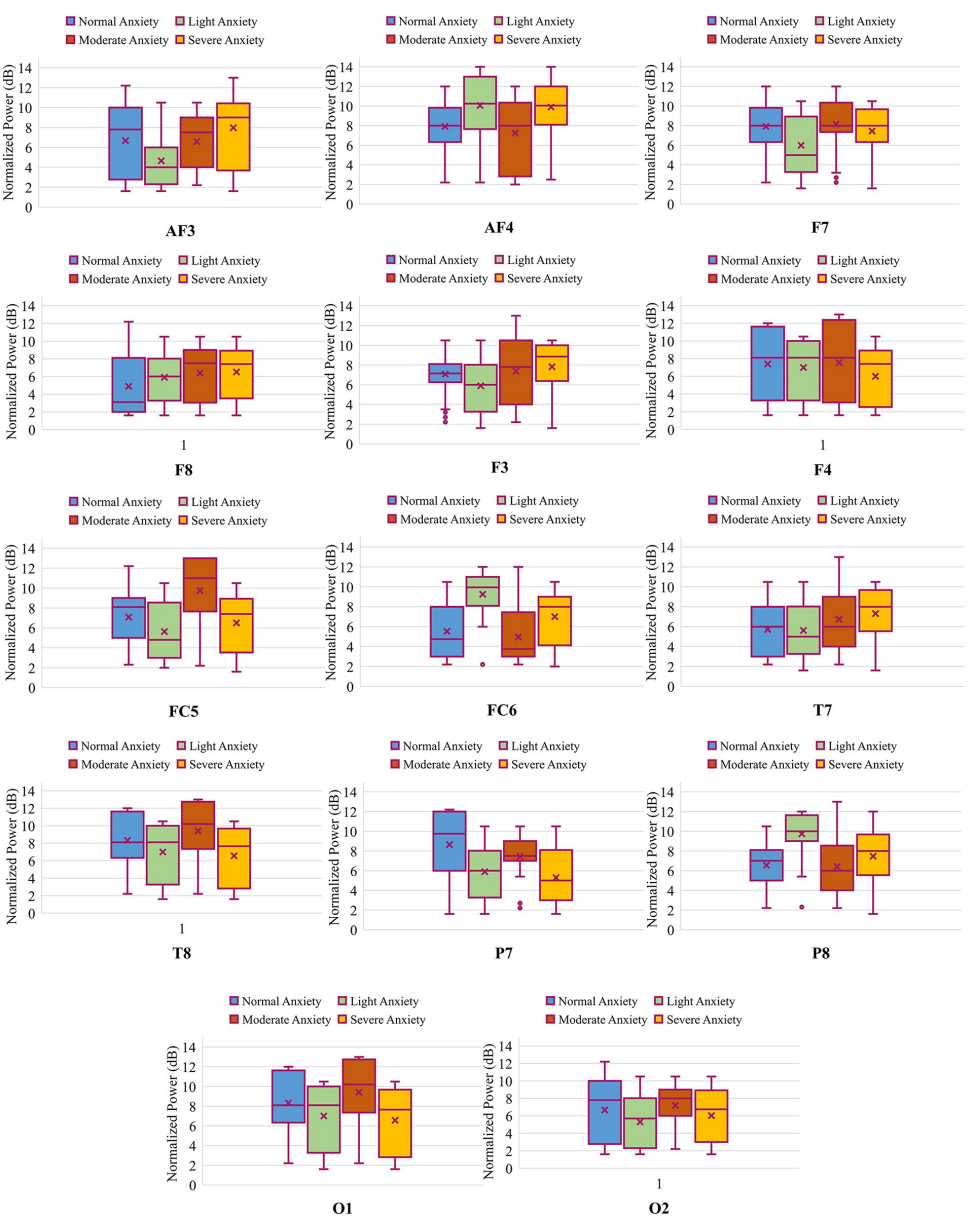
Box-and-whisker diagram of the power obtained from each channel of the Emotiv EPOC headset for four level anxiety classification.

Channel selection is conducted using student t-test for two-level and ANOVA for four-level anxiety classification. T-test and ANOVA are applied on the power spectral density (PSD) of the raw EEG data obtained from each channel of the Emotiv EPOC headset. PSD of the EEG data is computed using the Welch method with an overlapping window of 50%. The results of the statistical test based on both SAM and HAM-A rating reveals the fact that channels AF3, AF4, FC5, FC6, P7 and P8 are significantly different for both two (p-value of 0.017, 0.041, 0.039, 0.028, 0.030, 0.048, respectively) and four (0.035, 0.022, 0.029, 0.043, 0.019, 0.038, respectively) level anxiety detection. Apart from these six channels, no other channels of the Emotiv EPOC headset are statistically significant for either two or four-level anxiety detection. Figs 5 and 6 are the boxplots for normalized power spectral density (PSD) obtained from each channel of the Emotiv EPOC headset for two and four level anxiety classification, respectively. The normalized PSD is obtained using the following formula.

$$\begin{array}{c} {PSD}_{N}=\frac{\left(x_{i}-min\left(x\right)\right)}{\left(max(x)-min(x)\right)}*14, \end{array}$$ (1)

where *PSD*_*N*_ is the normalized power spectral density, *x*_*i*_ is the *i*^*th*^ sample of PSD, *min*(*x*) in the minimum value for PSD of a particular EEG channel and *max*(*x*) is the maximum value for the PSD of a particular EEG channel. It can be inferred from the graph that only the significantly different EEG channels have visually dissimilar patterns from each other for both two and four-level.

Feature extraction is performed from the selected subset of EEG channels. Mean power resulted in a total of 30 features from the five frequency bands of six selected channels of the Emotiv EPOC headset. Similarly, each of the RASM and AI feature groups resulted in a total of 15 features extracted from the EEG data of the selected channels of the Emotiv headset. Hence, the cumulative EEG feature vector obtained is composed of 60 values.

### 4.3 Performance analysis of EEG frequency band and feature selection

**Fig 7:**
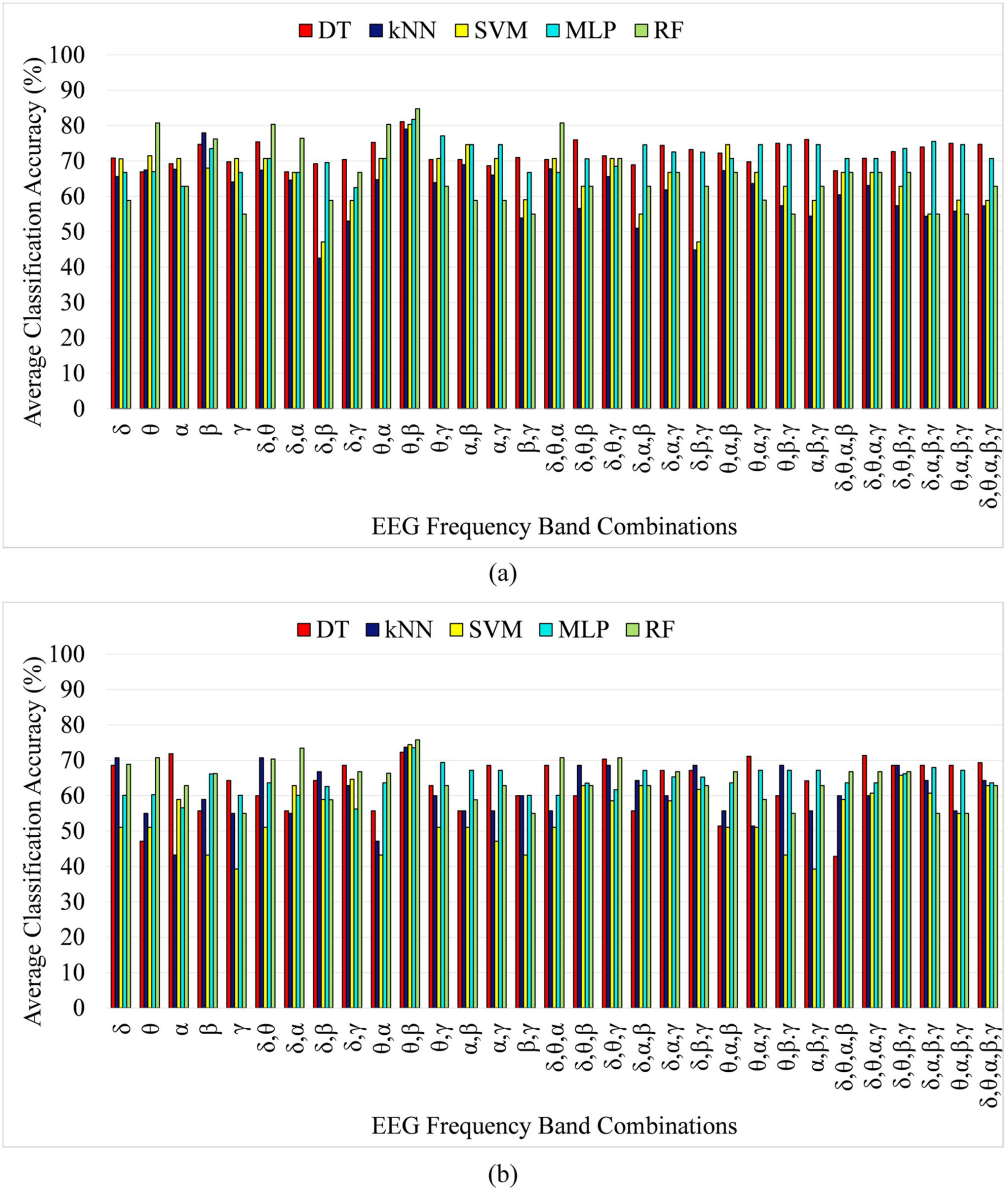
Classification accuracy of DT, k-NN, SVM, MLP and RF classifier using features from different frequency band combinations of EEG signal using SAM score based labeling for (a) Two-levels (b) Four-levels anxiety classification.

**Fig 8:**
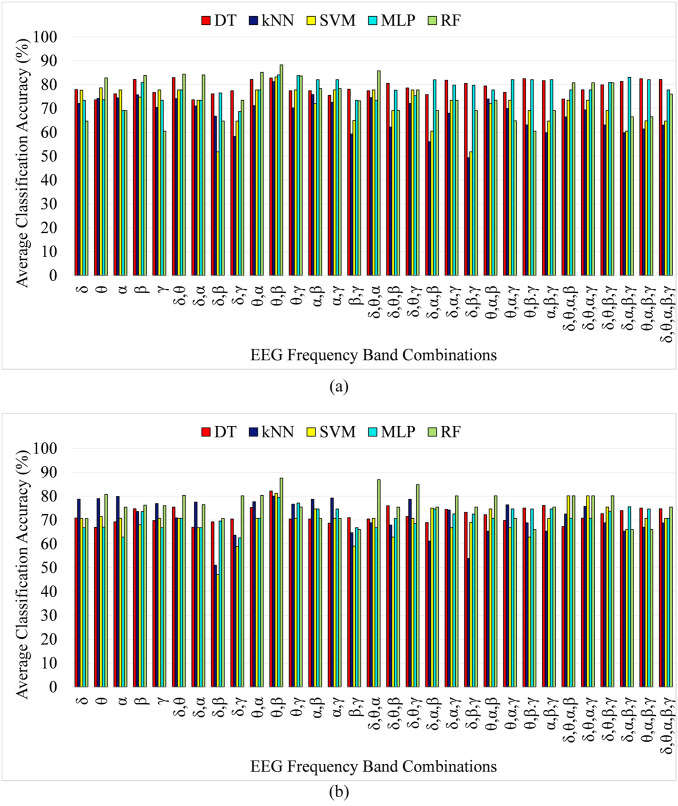
Classification accuracy of DT, k-NN, SVM, MLP and RF classifier using features from different frequency band combinations of EEG signal using HAM-A based labeling for (a) Two-levels (b) Four-levels anxiety classification.

One of the main contributions of this study is to identify the optimum subset of the EEG frequency band which results in the highest anxiety classification accuracy. To this end, the frequency band selection algorithm proposed in [81] was used to identify the appropriate subset of frequency bands. EEG frequency band selection is applied using a leave-one-out cross-validation scheme. Leave-one out cross validation method is a special type of cross-validation scheme in which the number of folds of the cross validation is equal to the number of instances in the dataset. Leave-one out cross validation treats each instance as a test data for once, while all other instances are considered as a training data. This phenomenon results in k number of models for k instances of data and the average of the classification accuracy obtained from each of the model is considered as the accuracy of the overall model. In the proposed anxiety classification, the number of instances is 4140 hence the value of k in cross-validation is 4140 in this experiment. In our study, it was found that using features only from the theta and beta band of the EEG data from the selected EEG channels resulted in the highest accuracy as compared to all other EEG frequency band combinations both for two and four class anxiety classification for SAM based labeling. Similarly, for HAM-A score-based labeling, the optimum subset of frequency bands include theta and beta band, resulting in the highest classification accuracy both for two and four-class labeling. Fig 7(a) and 7(b) shows the bar graph of the classification accuracy obtained from each of the EEG frequency band combinations using SAM-based labeling for two and four levels, respectively. Similarly, Fig 8(a) and 8(b) shows the classification accuracy using HAM-A based labeling for two and four levels of anxiety classification, respectively. The band selection algorithm resulted in a feature vector reduction from 60 (using features from all EEG frequency bands) to 18 (using features from only theta and beta band of EEG signal) features and accuracy of 84.77% and 75.77% for two and four-level anxiety classification, respectively using random forest classifier for SAM based labeling. Moreover, for HAM-A based labeling, the classification accuracy for two and four-level anxiety classification is 88.30% and 87.57%, respectively using the RF classifier. In order to achieve a classification model with low bias and low variance, hyperparameter tuning was applied and best parameters were chosen to find the classification model with the optimum complexity. The random forest classifier with a parameter of 500 decision trees with a depth of 32 levels resulted in best classification accuracy with an average bias and average variance of 0.022 and 0.039, respectively among all the classifiers. Moreover, the MALTAB used in the proposed scheme was version 2018a, running on a core i5 PC with 8GB RAM.

The extracted features from the selected EEG frequency bands are subjected to the feature selection phase via a recursive feature elimination technique to obtain the best subset of features from the selected frequency bands. The recursive feature selection algorithm when applied on two level anxiety classification problem using SAM based labeling, it resulted in a feature vector length of 10 consisting of MP (*β*_*AF*3_, *β*_*AF*4_, *β*_*P*7_, *θ*_*AF*3_, *θ*_*AF*4_, *θ*_*P*8_), RASM (*β*_*AF*3, *AF*4_, *θ*_*AF*3, *AF*4_), and AI (*β*_*FC*5, *FC*6_, *θ*_*FC*5, *FC*6_). Similarly, the selected subset of features for four level anxiety classification includes MP (*β*_*AF*3_, *β*_*FC*5_, *θ*_*FC*5_, *θ*_*FC*6_), RASM (*β*_*AF*3, *AF*4_, *θ*_*FC*5, *FC*6_), and AI (*β*_*AF*3, *AF*4_, *θ*_*FC*5, *FC*6_) resulting in a feature vector of length 8. For HAM-A based labeling, the selected subset of features for two level anxiety detection include MP (*β*_*FC*6_, *β*_*P*8_, *β*_*P*7_, *θ*_*AF*3_, *θ*_*FC*6_), RASM (*β*_*FC*5, *FC*6_, *θ*_*AF*3, *AF*4_), and AI (*β*_*P*7, *P*8_, *θ*_*FC*5, *FC*6_) resulting in a feature vector of 9. Moreover, the optimum subset of features from the selected frequency bands for four level anxiety detection using HAM-A based labeling constitutes a feature vector of length 10, which includes MP (*β*_*FC*6_, *β*_*P*8_, *θ*_*FC*5_, *θ*_*FC*6_, *θ*_*AF*3_), RASM (*β*_*AF*3, *AF*4_, *θ*_*AF*3, *AF*4_, *θ*_*FC*5, *FC*6_), and AI (*β*_*AF*3, *AF*4_, *θ*_*P*7, *P*8_).

### 4.4 Classification performance

Human anxiety classification into two and four-level is performed using five different machine learning algorithms i.e., k-NN, DT, RF, MLP, and SVM. Feature extraction is performed in MATLAB and the classification part of the algorithm is done using python environment. The results of the proposed scheme are evaluated using a leave-one-out cross-validation mechanism. An important thing to mention over here is that the training and test samples do not belong to the same subject. The proposed anxiety detection scheme is evaluated in terms of accuracy (Acc), F-value (F) and kappa statistics (K). The accuracy of a classification algorithm depicts the correct number of predictions from among the total number of predictions. Mathematically it is given as,

$$\begin{array}{c} Accuracy\;\;=\;\frac{TP+TN}{TP+TN+FP+FN}, \end{array}$$ (2)

where TP, TN, FP and FP represent the true positive, true negative, false positive and false negative respectively. F-value is the measure of the model accuracy on a given dataset. It provides a way to combine the precision and recall of an algorithm into a single measure. F-value is mathematically given as,

$$\begin{array}{c} F-value\;\;=\;2\;\times \;\frac{Precision\;\times \;Recall}{Precision\;+Recall}, \end{array}$$ (3)

where precision is given as,

$$\begin{array}{c} Precision\;\;=\;\frac{TP}{TP+FP}, \end{array}$$ (4)

and recall is given as,

$$\begin{array}{c} Recall\;\;=\;\frac{TP}{TP+FN}, \end{array}$$ (5)

Kappa statistics is the measure of the amount of agreement between two measurements. If the agreement between the two quantities is only by chance then kappa statistics have a value of 0 whereas, if a perfect agreement exists then kappa statistics gives a value of 1. Mathematically it is given as,

$$\begin{array}{c} Kappa\;\;=\;\frac{P_{observed}\;-\;P_{chance}}{1\;-\;P_{chance}},\; \end{array}$$ (6)

where *P*_*observed*_ is relative observed agreement among two human raters and *P*_*chance*_ is the hypothetical probability of chance agreement.

Table 1 presents the performance of the proposed anxiety classification scheme using both SAM and HAM-A based labeling methods. It is evident from the results that the highest classification accuracy is achieved for two and four-level anxiety classification using RF classifier and using both types of labeling. For SAM score-based labeling, the highest classification accuracy of 88.37% and 79.86% is achieved by RF classifier for two and four-level anxiety classification with a feature vector length of 10 and 8, respectively. Moreover, an F-value of 0.86 and kappa statistics of 0.84 are achieved for two-level and F-value of 0.78, and kappa statistics of 0.74 are attained for four-level anxiety classification. It can also be observed from the table that HAM-A based labeling provided better results for both two and four-level anxiety classification using random forest classifier as compared to SAM score-based labeling. Classification accuracy of 94.90% and 92.74% was achieved for two and four-level anxiety classification via HAM-A labeling with a feature vector length of 9 and 10, respectively. Moreover, an F-value of 0.93 and 0.90 and a kappa value of 0.88 and 0.86 is achieved for two and four-level anxiety classification, respectively.

**Table 1:** Performance comparison of proposed anxiety classification framework using both SAM and HAM-A based labeling for two and four levels in terms of different machine learning classifiers, Feature Vector Length (FVL), accuracy, F-value, and Kappa values (K).

Labeling Method	Anxiety Levels	Classifier	FVL	Accuracy (%)	F-value	Kappa values
SAM Score	Two	DT	12	82.42	0.80	0.78
SAM Score	Two	kNN	15	81.25	0.79	0.75
SAM Score	Two	SVM	13	82.34	0.81	0.79
SAM Score	Two	MLP	8	83.53	0.82	0.80
**SAM Score**	**Two**	**RF**	**10**	**88.37**	**0.86**	**0.84**
SAM Score	Four	DT	14	75.34	0.74	0.70
SAM Score	Four	kNN	7	76.45	0.74	0.70
SAM Score	Four	SVM	12	77.39	0.76	0.72
SAM Score	Four	MLP	17	76.59	0.75	0.71
**SAM Score**	**Four**	**RF**	**8**	**79.86**	**0.78**	**0.74**
HAM-A Score	Two	DT	19	85.39	0.84	0.82
HAM-A Score	Two	kNN	17	84.69	0.83	0.80
HAM-A Score	Two	SVM	11	86.98	0.85	0.82
HAM-A Score	Two	MLP	9	88.10	0.86	0.83
**HAM-A Score**	**Two**	**RF**	**9**	**94.90**	**0.93**	**0.88**
HAM-A Score	Four	DT	17	85.34	0.83	0.78
HAM-A Score	Four	kNN	14	83.48	0.82	0.77
HAM-A Score	Four	SVM	13	85.25	0.84	0.81
HAM-A Score	Four	MLP	9	82.43	0.81	0.75
**HAM-A Score**	**Four**	**RF**	**10**	**92.74**	**0.90**	**0.86**

Tables 2–5 presents the confusion matrices of two and four-level anxiety classification using SAM and HAM-A score-based labeling along with the precision and recall values. The confusion matrices augment the findings of Table 1 in which the RF classifier achieves the best results among all classifiers. In Table 2, 2300 out of 2640 instances are correctly classified for light anxiety class, whereas 1359 out 1500 severe anxiety instances are correctly classified using RF classifier. Table 3 presents the results for two-level anxiety classification using HAM-A score-based labeling. It can be observed from the table that 1145 out of 1260 light anxiety instances are correctly classified, whereas, 2784 out of 2880 instances for severe anxiety are correctly detected using the RF classifier. Table 4 presents the results for four-level anxiety detection using SAM score-based labeling. It can be observed from the table that 1907 out of 2340 normal anxiety instances, 248 out of 300 light anxiety instances, 132 out of 150 moderate anxiety instances, and 1031 out of 1350 severe anxiety instances are correctly classified using RF classifier. Similarly, Table 5 presents the findings for four-level anxiety classification using HAM-A score-based labeling. The table reports the fact that 330 out of 360 normal anxiety instances, 827 out of 900 light anxiety instances, 445 out of 540 moderate anxiety instances, and 2238 out of 2340 severe anxiety instances are correctly classified using RF classifier. Moreover, it can be noted from the confusion matrix tables that the highest precision and recall value is observed for RF classifier for all the anxiety classification cases.

**Table 2:** Confusion matrix for two level anxiety classification for DT, kNN, SVM, MLP and RF classifier using SAM based labeling.

LA	SA	Classified as	Recall	Precision
1980	68	LA = Light Anxiety	0.96	0.75
660	1432	SA = Severe Anxiety	0.68	0.95
(a) DT				
LA	SA	Classified as	Recall	Precision
2233	369	LA = Light Anxiety	0.85	0.84
407	1131	SA = Severe Anxiety	0.73	0.75
(b) kNN				
LA	SA	Classified as	Recall	Precision
2143	234	LA = Light Anxiety	0.90	0.81
497	1266	SA = Severe Anxiety	0.71	0.84
(c) SVM				
LA	SA	Classified as	Recall	Precision
2302	344	LA = Light Anxiety	0.86	0.87
338	1156	SA = Severe Anxiety	0.77	0.77
(d) MLP				
LA	SA	Classified as	Recall	Precision
2300	141	LA = Light Anxiety	0.94	0.87
340	1359	SA = Severe Anxiety	0.79	0.90
(e) RF				

**Table 3:** Confusion matrix for two level anxiety classification for DT, kNN, SVM, MLP and RF classifier using HAM-A based labeling.

LA	SA	Classified as	Recall	Precision
1032	377	LA = Light Anxiety	0.73	0.81
228	2503	SA = Severe Anxiety	0.91	0.86
(a) DT				
LA	SA	Classified as	Recall	Precision
1103	477	LA = Light Anxiety	0.69	0.87
157	2403	SA = Severe Anxiety	0.93	0.83
(b) kNN				
LA	SA	Classified as	Recall	Precision
907	186	LA = Light Anxiety	0.82	0.71
353	2694	SA = Severe Anxiety	0.88	0.93
(c) SVM				
LA	SA	Classified as	Recall	Precision
1032	264	LA = Light Anxiety	0.79	0.81
228	2616	SA = Severe Anxiety	0.91	0.90
(d) MLP				
LA	SA	Classified as	Recall	Precision
1145	96	LA = Light Anxiety	0.92	0.90
115	2784	SA = Severe Anxiety	0.96	0.96
(e) RF				

**Table 4:** Confusion matrix for four level anxiety classification for DT, k-NN, SVM, MLP and RF classifier using SAM based labeling.

NA	LA	MA	SA	Classified as	Recall	Precision
2003	12	13	127	NA = Normal Anxiety	0.92	0.85
137	251	8	267	LA = Light Anxiety	0.37	0.83
110	27	123	213	MA = Moderate Anxiety	0.26	0.82
90	10	6	743	SA = Severe Anxiety	0.87	0.55
(a) DT						
NA	LA	MA	SA	Classified as	Recall	Precision
1854	31	15	125	NA = Normal Anxiety	0.91	0.79
231	219	7	167	LA = Light Anxiety	0.35	0.73
127	27	122	133	MA = Moderate Anxiety	0.29	0.81
128	23	6	925	SA = Severe Anxiety	0.85	0.68
(b) kNN						
NA	LA	MA	SA	Classified as	Recall	Precision
1785	19	13	112	NA = Normal Anxiety	0.92	0.76
155	221	20	85	LA = Light Anxiety	0.45	0.73
21	29	100	55	MA = Moderate Anxiety	0.48	0.66
183	31	17	1098	SA = Severe Anxiety	0.82	0.81
(c) SVM						
NA	LA	MA	SA	Classified as	Recall	Precision
1709	12	6	106	NA = Normal Anxiety	0.93	0.73
329	270	4	87	LA = Light Anxiety	0.39	0.90
171	11	138	103	MA = Moderate Anxiety	0.32	0.92
131	7	2	1054	SA = Severe Anxiety	0.88	0.78
(d) MLP						
NA	LA	MA	SA	Classified as	Recall	Precision
1907	7	2	119	NA = Normal Anxiety	0.93	0.81
158	248	13	56	LA = Light Anxiety	0.52	0.82
142	27	132	144	MA = Moderate Anxiety	0.29	0.88
133	18	3	1031	SA = Severe Anxiety	0.87	0.76
(e) RF						

**Table 5:** Confusion matrix for four level anxiety classification for DT, k-NN, SVM, MLP and RF classifier using HAM-A based labeling.

NA	LA	MA	SA	Classified as	Recall	Precision
245	47	56	128	NA = Normal Anxiety	0.51	0.68
64	713	23	38	LA = Light Anxiety	0.85	0.79
15	64	443	42	MA = Moderate Anxiety	0.78	0.82
36	76	18	2132	SA = Severe Anxiety	0.94	0.91
(a) DT						
NA	LA	MA	SA	Classified as	Recall	Precision
295	77	21	61	NA = Normal Anxiety	0.64	0.81
43	695	44	107	LA = Light Anxiety	0.78	0.77
38	96	417	93	MA = Moderate Anxiety	0.64	0.77
19	32	58	2079	SA = Severe Anxiety	0.9+5	0.88
(b) kNN						
NA	LA	MA	SA	Classified as	Recall	Precision
250	72	23	90	NA = Normal Anxiety	0.57	0.69
33	703	43	58	LA = Light Anxiety	0.83	0.78
30	25	448	63	MA = Moderate Anxiety	0.79	0.82
47	100	26	2129	SA = Severe Anxiety	0.92	0.90
(c) SVM						
NA	LA	MA	SA	Classified as	Recall	Precision
300	37	27	97	NA = Normal Anxiety	0.65	0.83
17	693	38	103	LA = Light Anxiety	0.81	0.77
30	112	403	123	MA = Moderate Anxiety	0.60	0.74
13	58	72	2017	SA = Severe Anxiety	0.93	0.86
(d) MLP						
NA	LA	MA	SA	Classified as	Recall	Precision
330	21	27	12	NA = Normal Anxiety	0.84	0.91
7	827	16	51	LA = Light Anxiety	0.91	0.91
15	29	445	39	MA = Moderate Anxiety	0.84	0.82
8	23	52	2238	SA = Severe Anxiety	0.96	0.95
(e) RF						

### 4.5 Performance comparison and discussion

This section compares the proposed scheme with the other state-of-the-art methods available in the literature on the basis of the number of participants involved in the experiment, modalities, number of EEG electrodes, classifiers used, number of classes, and the accuracy achieved. The results are presented in Table 6 in which the methods used for the comparison of the proposed scheme include [39, 40, 42–45, 47–49]. In terms of the number of participants, our proposed scheme consists of EEG data from six trials of the experiment with 23 subjects, where each trial consisted of 30 seconds of data. In this study we have used 1 second of EEG data as an instance, therefore the total number of instances for 23 subjects becomes 4140 which is higher than all other available methods except [39] from which we have adopted the dataset. Secondly, in terms of the modality used, the studies involving only EEG data have achieved the highest accuracy of 93.95%, 87.18%, and 86.70% for two, three, and four-level anxiety classification, respectively. Our proposed scheme achieves higher classification accuracy of 94.90% and 92.74% for both two and four-level anxiety classification, respectively. Similarly, in comparison to multimodal studies, our scheme has a better performance both in terms of classification accuracy and computational complexity. Moreover, in terms of electrodes, only one EEG study presented in [44] used 1 electrode for the recording of EEG data but it only achieved an accuracy of 62.50% for three-level anxiety classification whereas, all other studies have used more electrodes than our proposed scheme. In comparison to this, our proposed scheme achieved a much higher accuracy for two and four-level anxiety classification using only 6 selected channels from among 14 channels of the Emotiv EPOC headset. Hence, it can be concluded from the results that the classification accuracy for anxiety detection can be significantly improved using channel selection before feature extraction and EEG frequency band selection before classification both for binary as well as multi-level anxiety detection.

**Table 6:** Performance comparison of the proposed framework with the state-of-the-art methods for human state anxiety classification.

Method, Year	Number of Participants	Modalities	Number of EEG channels	Classifier	Accuracy (Classes)
[39], 2021	23	EEG	14	SSAE	83.50% (2) 86.70% (4)
[42], 2021	34	EEG	32	SVM	87.18% (3)
[48], 2020	31	EEG	31	LR	77.01% (2)
[45], 2020	80	ECG, EDA, RR	–	Bagged tree	89.80% (2) 74.40% (3)
[49], 2019	30	GSR, BVP, ST	–	SVM	80.10% (4)
[43], 2018	59	HR	–	SVM	81.82% (2)
[47],2017	23	eye, mouth, head movement, HR	–	Adaboost	91.68% (2)
[40], 2017	16	EEG	57	NB	93.75% (2)
[44], 2016	20	EEG, PPG	1	kNN	62.50% (3)
**Proposed, 2021**	**23**	**EEG**	**6**	**RF**	**94.90% (2)** **92.74% (4)**

EDA = Electrodermal activity, RR = Respiration rate, BVP = Blood volumetric pressure, ST = Skin temperature, SSAE = Stacked sparse autoencoder, LR = Logistic regression, NB = Naive bayes

## 5 Conclusion

In this paper, a two and four-level anxiety classification framework is developed by using EEG data of 23 subjects recorded during exposure therapy and using Emotiv EPOC headset. Channel selection is performed to select the statistically significant channels using t-test for two-level and ANOVA for four-level on the power spectral density of the data from the EEG electrodes. Channels AF3, AF4, FC5, FC6, P7, and P8 are found to be statistically significant from among 14 channels of the Emotiv EPOC headset. Frequency domain features which include mean power, rational asymmetry, and asymmetry index are extracted from the selected EEG channels. Next, the frequency band selection algorithm is applied to identify the appropriate EEG frequency bands which in this study are theta and beta bands. The wrapper method is applied for feature selection from all the features of the selected frequency bands. Finally, the selected subset of features is fed into five different machine learning algorithms i.e., DT, kNN, SVM, MLP, and RF for anxiety classification. RF classifier achieved the highest classification accuracy both for two and four-level anxiety classification. An accuracy of 94.90% and 92.74% is achieved for two and four-level anxiety classification using HAM-A based labeling and RF classifier with a feature vector length of 9 and 10, respectively using data only from 6 selected EEG channels. Many clinical applications which can improve the quality of life of an individual in the society can be derived from the findings of the current study. The limitations of the proposed anxiety classification scheme include a lesser number of participants which restricts us from applying the deep learning model for classification. Moreover, the anxiety elicitation protocol was applied on participants with an average age of 30 years. The results of the experiment with subjects having higher average age e.g., around 50 years still need to be examined to further validate the findings of the proposed scheme. Furthermore, the effect of EEG data recorded from participants with different ethnical background also needs to be examined.
